# PROJECT SOAR PARTNERSHIPS: EXPLORING ADVANCE CARE PLANNING AMONG RURAL AFRICAN AMERICANS

**DOI:** 10.1093/geroni/igz038.1502

**Published:** 2019-11-08

**Authors:** Rebecca S Allen, Pamela Payne-Foster, JoAnn S Oliver, Christopher H Spencer, Deanne M Dragan

**Affiliations:** 1 The University of Alabama, Tuscaloosa, Alabama, United States; 2 The University of Alabama College of Nursing, Tuscaloosa, Alabama, United States

## Abstract

Sharing Opinions and Advice about Research (Project SOAR), funded by PCORI, trained individuals living in under-resourced and underserved communities how to evaluate and provide advice to scientists about recruitment procedures, survey items, and intervention components for implementation in their communities. In partnership with the HELLO Project (Van Scoy, Green, & Volpe, 2019), Project SOAR community partners recruited 50 rural African American adults to consider their values, plans, and treatment preferences near the end of life while playing the HELLO game. Community and research partners along with a HELLO Project representative facilitated questionnaire completion and the process of the HELLO game to promote advance care planning. Tables of 10 participants held facilitated discussions for each HELLO game item and recorded their individual wishes and plans in HELLO booklets. Community participants had difficulty completing questionnaires without assistance; however, all were engaged and accepting of game-like discussions regarding advance care planning.

